# Evaluating the completeness of electronic health records in dental education: a big data study

**DOI:** 10.3389/froh.2025.1535164

**Published:** 2025-06-03

**Authors:** Tamanna Tiwari, Maxim Kondratenko, Nihmath Nasiha, Toan Ong, Sangeetha Chandrasekaran, Gary Kostbade, Zachary Giano

**Affiliations:** ^1^ School of Dental Medicine, University of Colorado, Aurora, CO, United States; ^2^ Department of Pediatrics, University of Colorado Anschutz Medical Campus, Aurora, CO, United States; ^3^ Center for Innovative Design and Analysis, University of Colorado Anschutz Medical Campus, Aurora, CO, United States

**Keywords:** electronic health record, BigMouth dental data repository, completeness, behavioral health, dental workforce

## Abstract

**Objectives:**

The BigMouth Dental Data Repository is an oral health database developed from de-identified electronic health record (EHR) data from eleven dental schools within the United States. To better understand how this database can be used for further research, the repository must be analyzed for data quality, such as accuracy, consistency, and completeness. This study determined the completeness of all patient health records between 2017 and 2019, including demographic, dental, behavioral, and health history variables at the students, faculty, and resident level.

**Methods:**

This study analyzed demographic (age, gender, race/ethnicity, zip code, insurance), dental (pain ratings), behavioral (tobacco, alcohol, and drug use), and health history variables for completeness. ANOVA was conducted to detect differences in providers collecting data by year (using Tukey *post hoc* differences at *p* < .05). Effect sizes are presented by comparing students to all other provider types.

**Results:**

Overall, the data showed high completeness in demographic variables (97.6%-99.9% for age, gender, and zip code) among the total sample of 543,363 patient visits. However, lower completeness rates were found in dental and behavioral variables (ranging from 1.5% to 66.1%), suggesting potential limitations for certain research applications. The study found significant differences in the completeness of records between students, faculty, and residents. In demographic variables, students demonstrated significantly higher completeness rates than faculty across the years 2017–2019, with 79.8%, 79%, and 78.8% completeness for race/ethnicity records, respectively. Furthermore, residents and faculty exhibited significantly higher completeness rates (76.8% and 86.7%, respectively) in insurance information compared to students (56.7%). Notably, students showcased greater completeness percentages in variables related to tobacco use, alcohol use, drug use, and health history compared to both faculty and residents.

**Conclusion:**

This study underscores significant variations in the completeness of EHR data among students, faculty, and residents across different schools. Despite these variances, the overall findings suggest a robust level of completeness in the demographic and health variables within the dataset.

## Introduction

One of the critical components of dental education today is teaching our students to manage their practice and learning to use and manage electronic health records (EHR) comprehensively. The replacement of paper health record systems with electronic health records increases the capability for more comprehensive and accessible documentation, leading to higher quality care and improved population health (1). A recent study has shown that using EHR during dental education correlated positively with the development of students, critical thinking skills and promoted greater ease for students in obtaining data from patients (2). In addition, some features of EHRs assist medical and dental providers in reducing errors and ensuring patient welfare in healthcare settings (3). For example, patient records are less likely to be lost or damaged. Retrieving patient information is faster and more reliable. EHRs often are embedded with clinical decision support systems. A clinical decision support system compares unique patient information with guideline algorithms to generate recommendations and alerts for providers – such as a system that can evaluate potential drug interactions and allergies, reducing prescription errors and improving the quality of patient care (4, 5).

Data quality, a multidimensional concept encompassing completeness, accuracy, and consistency, is crucial for drawing reliable research conclusions, with completeness and accuracy often being prioritized in health research (6).

Proper EHR usage varies significantly in academic dentistry. A 2021 study developed a measure to determine the completeness of periodontal disease documentation across four dental institutions (5). Despite using the same EHR and standardized diagnosis terminology, scores between the involved institutions varied between 0.97% and 99.49% (7). Another study compared the Dental Diagnostic System (DDS) used in nearly 10 million procedures between academic and private dental sites (6). The two measurements that were considered were utilization (instances that diagnoses are documented in a structured format) and validity (frequency of valid pairs i.e., accurate matching of diagnostic terms with their corresponding treatment procedures divided by the number of all treatment codes entered). Over the four-year observation period, the academic institutions and private practices significantly improved in both categories, including a 1.5-fold increase in utilization. Private dental sites had overall higher scores, although academic dental sites documented a higher proportion of diagnoses associated with orthodontic and restorative procedures. This is an example of how EHR completeness are dependent on context and can vary between existing and expected dental providers (8).

Most students consider EHRs to be a helpful tool that facilitates the organization, accessibility, and overall documentation of patient information. They also report that electronic prompts lead them to collect more complete patient histories when using EHRs (9). However, despite the growing necessity and use for EHR competency, students and residents may not receive adequate or consistent EHR training (10). Also, certain clinical establishments may have limitations on trainee's access to electronic health records (EHRs) (9). Some research has shown that students could prioritize billing compliance over thorough patient care documentation, leading to the propagation of inaccuracies in medical records (11).

As the use of EHR is increasing in dental schools and dentistry overall, research needs to be done to evaluate the quality of the data to improve oral health outcomes. In addition, necessary advancements must be made to standardize measurements, reporting, and training to realize the full benefits of these tools. Understanding the level of completeness in an EHR database can improve the validity and reliability of EHR and ultimately can improve patient care and dental education.

This study assessed the completeness of patient records from 2017 to 2019, specifically targeting demographic, dental, behavioral, and health history variables at the student, faculty, and resident levels within the BigMouth database to understand its potential for further research better. Completeness encompasses the degree and nature of missing values within the EHR-based database (12). It can be measured at different granularities and dimensions: the record of the patient as a whole or its logical components, the record of the patient over time, and using the intrinsic requirement of a patient record being complete i.e., completeness of variables of interest recorded during the clinical visit (13). The National Institutes of Health define completeness as the “presence of necessary data”; others have defined it as “fitness of data for research use” (14, 15). We hypothesized that the completeness of the EHR data would vary at provider-level (faculty, residents, and students).

## Methods

This study was approved by the Colorado Multiple Review Board.

### Data source

Data for this study were derived from the BigMouth dental data repository [https://www.uth.edu/bigmouth/] housed at the University of Texas at Houston (16). This is one of the largest data repositories of oral and systematic health information comprised of de-identified EHR data containing demographics, medical, and dental records, including dental codes, procedures, medication history, and self-reported overall health of over 4.5 million patients. This repository, which was established in 2012 by four dental schools, is an aggregation of the data collected by students and faculty in student clinics and faculty practice, respectively, from 11 dental schools. Approval is gained from the BigMouth data review group, which has representatives from each participating dental school before data extraction. Furthermore, the participating schools in this study were anonymized by letter (e.g., institution A) by the BigMouth data management team. All eleven dental schools use Axium software to document patient data into the EHR, which in turn is deposited into the BigMouth repository.

### Data extraction

Data for this study were extracted from the BigMouth repository for 2017, 2018, and 2019. Data completeness was defined as any answer under each variable, i.e., an answer of yes, no, prefer not to answer, and I don't know constituted complete. If none of these responses were present, it was considred missing. Importantly, the evaluation of completeness of data was conducted separately for the data from each individual year. Additionally, completeness was assessed based on whether there was a recorded answer for each variable within a participant's record, without consideration of the specific date within the year.

Several dental schools in the United States utilize the axiUm EHR platform, a commercial software acquired by Henry Schein in 2012 (17). axiUm is customizable according to the needs of every school; thus, variabilities in data can be seen present. To make sure that variabilities were reduced several steps were taken. Data mapping was done to synchronize the data categories before extraction of data. Oral health evaluation codes were used (D0120 = periodic oral evaluation – established patient, D0140 = limited oral evaluation – problem focused, D0150 = comprehensive oral evaluation – new or established patient, D0160 = detailed and extensive oral evaluation – problem focused, by report, D0170 = re-evaluation - limited, problem focused (established patient; not post-operative visit, D0180 = comprehensive periodontal evaluation – new or established patient, D0190 = screening of a patient) as these codes would include the variables needed for the study. The following variables were extracted: demographic (age, gender, zip code, race/ethnicity, insurance), dental (pain rating), behavioral (tobacco, alcohol, and drug use), and health history (if the patient reported any medical history such as diabetes, hypertension,). These attributes were chosen as there was the least variability in the data from different institutions, and thus, data mapping was simpler to complete. At the provider level, the data extracted were categorized as student, faculty, resident, and others group (other group included hygienist, non-dental provider, off-site provider, or non-specified). Our main objective was to compare the differences between the student, resident, and faculty groups so any other provider was grouped as “other.”

### Data analysis

First, frequencies were determined for all variables. These include demographic variables (age, gender, race/ethnicity, insurance), dental variables (pain rating), behavioral variables (tobacco, alcohol, drug use) and health history variable. Valid data (data present) was coded as 1, with missing data coded as 0 in order to estimate the relationship between the frequencies of missing and complete data for race/ethnicity by institution. This procedure was repeated for institution and provider type to compute the possible association between their missing and complete data for race/ethnicity data frequencies.

Next, an ANOVA was conducted to detect differences in providers' collection of data (used as the independent variable coded as a student, faculty, resident, or other) for the following dependent variables: behavioral variables (tobacco/drug/alcohol use), health history, and pain rating (using Tukey *post-hoc* differences at *p* < .05). Results were stratified by year.

The data was analyzed at the level of provider type (students, faculty, residents), aggregating information across multiple patient visits. Although multiple data points may come from the same patient, these data points were treated as independent for the purpose of assessing differences in completeness rates between provider types. The primary goal of this study was to compare the overall performance (i.e., completeness rates) of different provider groups rather than individual patient outcomes, thus justifying the use of ANOVA. It should be noted that although our dependent variables were dichotomous, an ANOVA has been shown to prove an appropriate statistical technique when the dependent variable is dichotomous in fixed effect models. The degree of freedom for error is at least 40 (18). The Tukey *post-hoc* test assessed pairwise differences between provider groups, assuming that the overall ANOVA model was significant. Since our main goal was to determine whether significant differences existed between specific provider types (e.g., students vs. faculty), the Tukey test provided a method to explore these differences in a controlled manner while adjusting for multiple comparisons.

Effect sizes (Cohen's d) are presented by comparing students to all other provider types. Effect sizes, such as Cohen's d denoted by the letter “d”, provide standardized measures to quantify the magnitude of differences between groups or the strength of relationships between variables, allowing for meaningful comparisons across studies and contexts. Effect sizes were interpreted as follows: small (d = 0.20), medium (d = 0.50), and large (d = 0.80). These values indicate the magnitude of the effect, with larger values suggesting a stronger effect.

## Results

The total number of visits across all three years is 543,363 (used in all tables/analyses), with 380,406 patients. Table 1 presents the results of variable completeness by demographic, dental, and behavioral variables stratified by year (aggregate completeness by year is also presented). It is important to note that these percentages do not change regardless of analyzing either the number of visits or the number of patients, as patient data is rolled over across visits. For example, if a patient's demographic information is collected, these data are included in their next visit. Among demographic variables, age, gender, and zip code showed high rates of completeness (97.6%–98.1%), while lower levels of completeness were found in race/ethnicity (71.9%–73.2%) and insurance type (65.3%–68.3%). The dental variable of pain rating was found to be among the least complete, ranging from 1.5% to 2.0%. Behavioral variables widely varied, with the most complete being health history (63.0%–66.1%), followed by alcohol use (42.6%–48.3%), drug use (35.6%–38.4%), and tobacco use (27.1%–34.9%).

**Table 1:** Demographic, dental, behavioral, and health history Variable completeness by year.

EHR variables	2017 (%)	Number of patients	2018 (%)	Number of patients	2019 (%)	Number of patients	2017-2019 (%)
Demographic variables							
Age	97.6	167,990	98.0	196,175	98.1	181,198	97.9
Gender	99.9	167,990	99.9	196,175	99.8	181,198	99.9
Race/ethnicity	73.1	167,990	73.2	196,175	71.9	181,198	72.7
Zip code	99.9	167,990	99.9	196,175	99.9	181,198	99.9
Insurance	66.5	167,990	65.3	196,175	68.3	181,198	66.7
Dental variables							
Pain rating	1.5	167,949	1.7	196,175	2.0	181,198	1.7
Behavioral variables							
Tobacco use	27.1	167,990	34.9	196,175	33.7	181,198	32.1
Alcohol use	42.6	167,990	48.3	196,175	44.6	181,198	45.3
Drug use	38.4	167,990	36.1	196,175	35.6	181,198	36.6
Impression of Health	63.0	167,990	66.0	196,175	66.1	181,198	65.1

Total of total patient visits (2017–2019) = 543,363.

**Figure 1:**
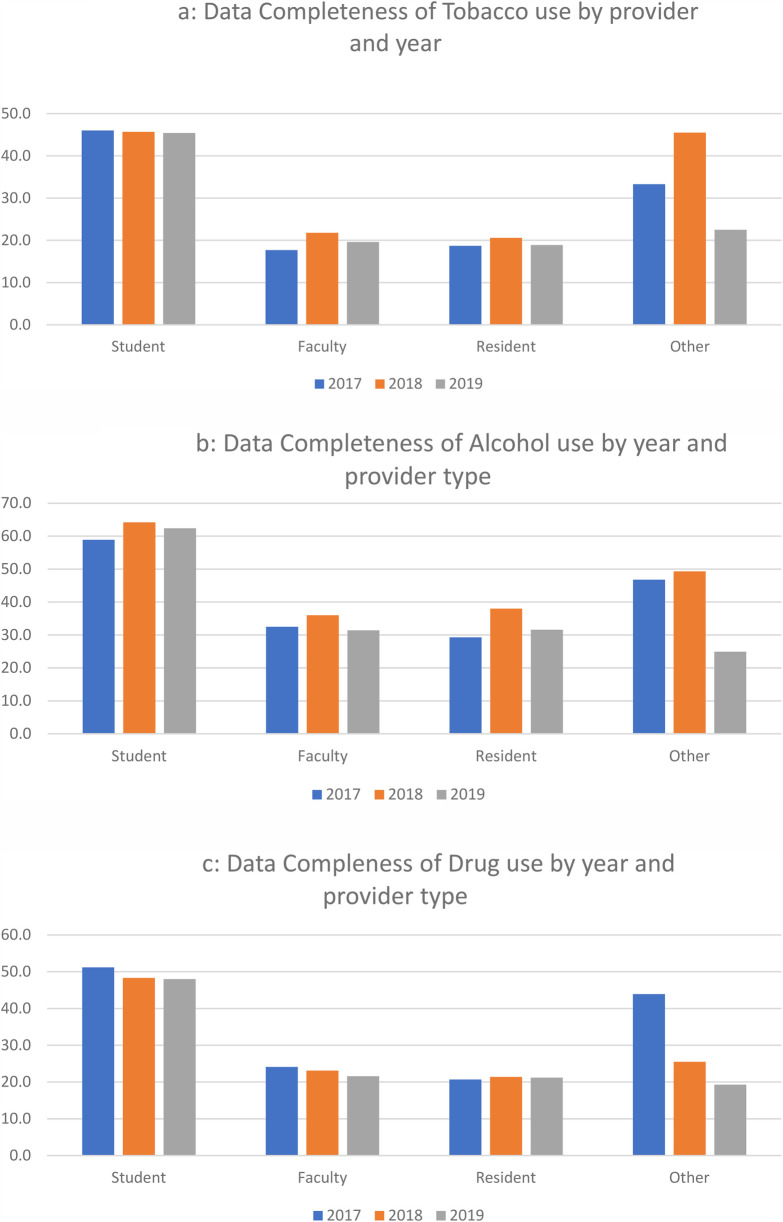
**(a)** Data completeness of tobacco use by provider and year. **(b)** Data completeness of alcohol use by year and provider type. **(c)** Data completeness of drug use by year and provider type.

Table 2 presents the results of each individual variable's completeness by provider type, stratified by year. For better visualization, we have provided Figures 1a–c for the completeness of tobacco use, alcohol use, and drug use. For tobacco use, residents and faculty had the lowest aggregate average completeness (19.5% and 19.8%, respectively), while students had the highest rate of completeness (45.7%). Those classified as other had 33.4% complete. It should be noted, however, that the range for other providers varied the most, ranging from 22.5% (2019) to 45.5% (2018), a 23-percentage point difference (the next greatest percentage point difference by year was 4.1% by faculty). Statistically significant differences were found among all combinations of provider type except for faculty vs. residents. Effect sizes for students vs. faculty, residents, and other providers are also shown. While students compared to other had a small effect (*d* = .19), students compared to faculty and residents had a medium effect size (*d* = .49 and .51, respectively).

**Table 2:** Completeness of behavioral variables by providers for the years 2017–2019.

Tobacco use	2017 (%)	2018 (%)	2019 (%)	2017–2019 (%)	Sig. Diff. (2017–2019)
Student	46.0	45.7	45.4	45.7	All, except for Faculty vs. Resident
Faculty	17.7	21.8	19.6	19.8	
Resident	18.7	20.6	18.9	19.5	
Other	33.3	45.5	22.5	33.4	
Effect sizes					
	Faculty	Resident	Other		
Student	0.49	0.51	0.19		
Alcohol use					Sig. Diff. (2017–2019)
Student	58.9	64.2	62.4	61.9	All, except for Faculty vs. resident
Faculty	32.5	36.0	31.4	33.3	
Resident	29.3	38.0	31.6	33.4	
Other	46.8	49.3	24.9	39.2	
Effect sizes					
	Faculty	Resident	Other		
Student	0.52	0.49	0.38		
Drug use					Sig. Diff. (2017–2019)
Student	51.2	48.3	48.0	49.1	All
Faculty	24.1	23.1	21.6	22.9	
Resident	20.7	21.4	21.2	21.2	
Other	43.9	25.5	19.3	27.8	
Effect Sizes					
	Faculty	Resident	Other		
Student	0.56	0.61	0.47		

Total *N* = 543,363.

Similar to tobacco, students had the highest aggregate rate of completeness for alcohol use (61.9%), followed by other providers (39.2), residents (33.4%), and faculty (33.3%), with statistically significant differences in all combinations except for faculty vs. residents. Effect sizes for students compared to faculty, residents, and others were medium (*d* = .52, .49, and .38, respectively).

The same patterns were found in drug use (albeit with generally lower rates compared to tobacco), with students having the highest rate (49.1%), other providers (27.8%), faculty (22.9%), and residents (21.2%). All groups were significantly different from one another, with effects sizes for students compared to faculty, residents, and others being medium (*d* = .56, .61, and .47, respectively).

With respect to health history (Table 3), students also had the highest rates for health history completion (76.6%), followed by other providers (72.8%), faculty (59.1%), and residents (40.2%), with all groups being significantly different from all other groups, respectively. While the effect size for students compared to residents was large (*d* = .79), and compared to faculty was medium (*d* = .38), the effect size compared to others was small (*d* = .08).

**Table 3:** Completeness of health history Variable by providers for years 2017–2019.

Provider	2017 (%)	2018 (%)	2019 (%)	2017–2019 (%)	Sig. Diff. (2017–2019)
Student	74.3	77.5	77.9	76.6	All
Faculty	56.3	60.6	60.1	59.1	
Resident	38.3	41.5	40.7	40.2	
Other	79.5	83.1	59.0	72.8	
Effect sizes					
	Faculty	Resident	Other		
Student	0.38	0.79	0.08		

Total *N* = 543,363.

Table 4 shows the results for completeness by pain rating by year. The pain rating was the only variable where faculty had the highest completeness rate compared to all others (3.5%), followed by students (1.6%), residents (.3%), and other providers (.1%). Faculty completeness rates were significantly different from all others, while students were significantly different from residents and other providers. Effect sizes for students compared to faculty, residents, and other were small (d = .12, .13, and .17, respectively).

**Table 4:** Completeness of pain ratings by providers for years 2017–2019.

Provider	2017 (%)	2018 (%)	2019 (%)	2017–2019 (%)	Sig. Diff. (2017–2019)
Student	1.3	1.5	1.8	1.6	Faculty > all Student > resident, other
Faculty	3.1	3.6	3.8	3.5	
Resident	0.4	0.3	0.3	0.3	
Other	0.0	0.0	0.1	0.1	
Effect sizes					
	Faculty	Resident	Other		
Student	0.12	0.13	0.17		

Total *N* = 543,363.

**Figure 2:**
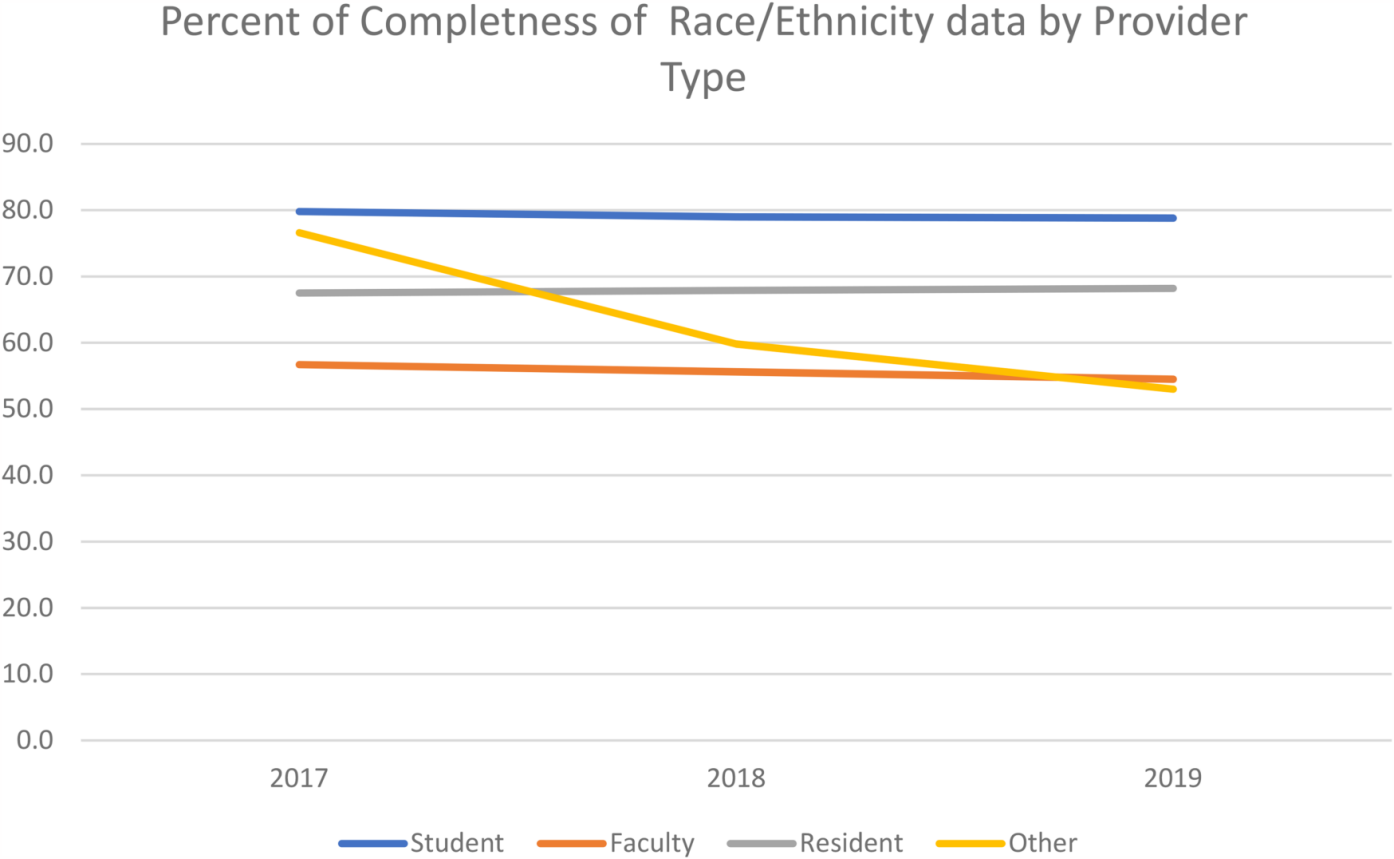
Percent of completeness of race/ethnicity data by provider type.

Table 5 and Figure 2 present the results of completeness of race/ethnicity by provider. While the other category had the highest rate in 2017 (86.7%), students had higher rates in both 2018 and 2019 (80.2% and 79.3%, respectively), as well as overall across all three years (80.0%). Effect sizes for students compared to faculty, residents, and others varied (d = .47, .25, and .21, respectively).

**Table 5:** Completeness of race/ethnicity by providers for years 2017–2019.

Provider	2017 (%)	2018 (%)	2019 (%)	2017–2019 (%)	Sig. Diff. (2017–2019)
Student	80.4	80.2	79.3	80.0	All
Faculty	59.0	59.5	57.8	58.8	
Resident	68.0	69.5	68.7	68.8	
Other	86.7	68.2	62.6	70.8	
Effect sizes					
	Faculty	Resident	Other		
Student	0.47	0.25	0.21		

Total *N* = 543,363.

## Discussion

For healthcare providers, a complete data collection helps them understand their patients comprehensively and provide individualized care, and teaching skills in data collection is critical. Therefore, it is essential to analyze the extent to which all patient data is collected.

Complete EHR data is essential as a core component of dental education, patient safety, understanding trends, and assessing oral health equity (19). In dental education, complete EHR data assists with decision-making in treatment planning and implementing an evidence-based education (20). EHR systems also have the potential to improve patient safety and quality of care by enhancing the quality and quantity of information available to providers for decision-making. The data stored in large databases can also be compared over time for aggregated populations and sub-populations of individual patients. Complete EHR data can be analyzed to provide information about population health trends. Additionally, we need complete EHR data to understand if healthcare disparities are being reduced. The following complications can arise from incomplete EHR data:

Healthcare providers may make assumptions leading to treatment errors, compromising patient health outcomes and safety. For instance, the absence of drug therapy alerts can have negative consequences for patients (21).

Health system managers may underestimate compliance with standards of care.

Researchers: The prevalence of disease may be underestimated, which can impact further research strategies.

Race and ethnicity data are among those that are commonly missed and inconsistent due to variability in data collection (22, 23). Variations in administrative systems among institutions, coupled with differences in data entry practices, documentation standards, and system configurations, can lead to gaps and disparities in data completeness. Additionally, the variability observed may stem from differences in the place of data collection and the individuals responsible for data capture, highlighting the need for standardization across healthcare organizations to ensure consistent and reliable data for research and decision-making purposes. Despite these challenges, demographic variables such as age, gender, and zip code exhibited high completion rates (97.6%–99.9%) among the total sample of 523,857 participants. However, completeness rates were lower for dental and behavioral variables (ranging from 1.5% to 66.1%), indicating potential limitations for certain research applications. While demographic data show good completeness, there is a need for improved standardization to ensure consistent and reliable data across healthcare organizations for research and decision-making purposes.

Research has shown that foreign-born or non-English speaking patients may find it difficult to answer the questionnaire accurately, while other patients may have reservations about answering culturally sensitive questions (22). The data in this study, which is provider-collected data, may be subjected to person-to-person variation in data collection and data entry errors that may further lead to incomplete data. However, student completeness of the race/ethnicity variable (79.2%) was higher than all other groups. This can be attributed to several factors, including oversight from faculty, as completing the data may contribute to students' competencies or grades. Additionally, several software used in dental education have built-in checks that flag incomplete entries, preventing students from proceeding with treatment until all required demographic information is provided. These measures help ensure that the necessary data is collected before treatment begins. Also, higher student completeness of race/ethnicity data demonstrates that students are learning to communicate about difficult questions with patients, as asking about race/ethnicity can be a difficult conversation, sometimes (17).

Comprehensive dental and health histories for each patient help understand how to individualize patient care. Regarding dental history, pain ratings help determine emergent dental care needs. However, the overall completion of this variable was 1.7% between 2017 and 2019. Furthermore, the average completeness of health history varied between 45 and 96.4% between different schools. However, students had significantly higher completeness (76.6%) as compared to other group (72.8%), faculty (59.1%), and residents (40.2%). Overall, there is a need for improvement in a complete collection of both health and dental histories for each patient. There is some evidence that some large organizations have devised standardization protocols for achieving data completeness (24). Setting a high standard by meticulously collecting data elements to ensure comprehensive patient care and effective disease prevention and treatment strategies. A level of data completeness not only enhances clinical decision-making but also facilitates proactive patient care, evidence-based prevention, and treatment methods. In addition, a complete EHR can help to standardize clinical workflows and implement robust data analytics, helping dental schools move closer to achieving the quadruple aim of better health, better care, lower cost, and an engaged workforce, ultimately improving oral health outcomes for patients (24).

Dental students have the opportunity as front-line health professionals to screen for substance abuse. For instance, some interprofessional education programs have been involving students in Screening, Brief Intervention, and Referral to Treatment (SBIRT) training to improve screening efforts (25). Therefore, collecting patient behavioral data such as tobacco, alcohol, and drug use is essential to provide comprehensive care. Overall, students had higher percent completion for tobacco, alcohol, and drug use (45.7, 61.9, and 49.1%, respectively) as compared to all other groups. However, the completion of these categories widely varied by institution, showing a difference in the emphasis on understanding these habits by school. Also, we have less information on the training provided to students and residents in this area and how that supports data collection. Regardless, data collection of social and behavioral variables can lead to a better understanding of health outcomes and ways to address underlying health disparities better (26).

The study results were discussed with the BigMouth review committee, and representatives were provided these results so they could take them back to their institutions and discuss strategies to improve data collection. Most institutional representatives spoke about the data collection process for faculty and pointed out that it was mainly done by staff within the clinic rather than the faculty themselves. Thus, the missingness of data was higher. Although the process is similar at most institutions, it should not exempt faculty from standardized processes. One of the ways to reduce missing data at every level (especially for faculty and staff) is to expose them to effective and repeated training, clinical decision support, regular audits and feedback, completeness indicators and alerts, and incentives to help improve data collection rates within dental schools. Such discussions have been conducted several times within the BigMouth review committee. However, the repository does not have any authority to make any changes for other institutions; it can inform them about the completeness of their data.

Research has shown that the EHR incentive plans to capture race, ethnicity, and language data completely and accurately from EHRs encouraged providers to demonstrate meaningful use of the EHRs and allowed for significant improvements in accurately capturing this information (27). Lee et al (27) recommend the ABC plan as one of the ways to overcome the challenges of capturing race/ ethnicity information. “A” of the plan involves Adjusting the EHR system specifically to replace “unknown” in the race and ethnicity fields with “Refused/Don’t know” and also allows reporting of multiple races. “B” of the plan involves Building awareness among all professionals and patients to emphasize the rationale and importance of collecting this information. “C” involves collaboration and sharing lessons learned with other health systems to identify common challenges and possible solutions. More such research and recommendations are needed in the future to overcome the challenges revolving around the collection of race/ethnicity data and to standardize the race/ethnicity categories (27).

The study has a few limitations. As with all big data studies, the researchers have to rely on the data as it was provided to us; data collection was done before this study, so no changes can be made to it. Also, as this data is de-identified, it is not possible for the researchers to find the institutions the data belongs to. While the researchers were able to compare institutions based on anonymized letters, the limitation arises from the inability to attribute specific data points to individual institutions due to the de-identification process. Although comparative analyses between institutions were feasible, the lack of identifiable information restricts the ability to trace specific data points back to particular institutions for deeper investigation or validation. Therefore, while broad comparisons and analyses across institutions were possible, the lack of identifiable data limits the granularity and specificity of the findings.

## Conclusion

In conclusion, this study underscores the role of comprehensive data collection in the clinical environment and its profound impact on dental education. As academic models increasingly pivot towards person-centered care, the cornerstone lies in thoroughly gathering data. This enables students, residents, and faculty to grasp a holistic understanding of their patients, empowering them to make informed clinical decisions based on a diverse array of factors. However, the study reveals deficiencies in data collection across various dimensions highlighting the pressing need for enhanced training and enforcement of patient data collection protocols within dental institutions.

## Data Availability

The raw data supporting the conclusions of this article will be made available on request to the BigMouth Dental Data repository. Please contact the corresponding author for further details.
